# Tumor regression in rectal cancer after intensified neoadjuvant chemoradiation: a morphometric and clinicopathological study

**DOI:** 10.1186/s12957-015-0572-z

**Published:** 2015-04-21

**Authors:** Friedrich Prall, Oliver Schmitt, Leif Schiffmann

**Affiliations:** Institute of Pathology, Rostock University, Strempelstraße 14, D-18055 Rostock, Germany; Institute of Anatomy, Rostock University, Gertrudenstraße 11, D-18055 Rostock, Germany; Department of Surgery, Rostock University, Schillingalle 70, D-18055 Rostock, Germany

**Keywords:** Rectal cancer, Neoadjuvant radiochemotherapy, Regression grading, Morphometry

## Abstract

**Background:**

High interobserver variation is a well known drawback of conventional tumor regression grading, and reaching consensus among pathologists may require a considerable effort. Therefore, in this study, morphometry was tried to assess tumor regression, and its prognostic role was explored.

**Methods:**

Tumor regression was quantified by a point counting method to yield tumor area fraction (TAF) as an index of remaining vital tumor.

**Results:**

In a series of 104 patients with clinically advanced rectal cancer treated with neoadjuvant radiochemotherapy, TAFs were distributed continuously towards complete regression which was observed in 8.7% of the cases. Plotting TAFs grouped by a conventional regression grading (Dworak’s) revealed considerable overlap between groups. In a control series of untreated cancers, only TAFs of cancers with an expansive invasive border were setoff clearly from TAFs obtained for the study cases, but TAFs of control cases with an infiltrative invasive border and mucinous carcinomas extended well into the range of TAFs recorded for regressing tumors. Locoregional recurrence (*N* = 10) was significantly associated with perineural tumor infiltration and capsule transgressing lymph node metastasis/tumor deposits but not with the degree of tumor regression. Overall survival was better for patients with major regressions (≤20th percentile by morphometry, or Dworak regression grade (DRG) 4/5), although statistical significance was not reached.

**Conclusions:**

Morphometry of tumor regression is feasible and explains why conventional regression grading is so difficult to perform. Assessment of tumor regression, by subjective grading or morphometry, does not appear to convey major prognostic information, at least not substantially beyond histopathological tumor staging. This observation discourages expending too much effort on developing this aspect of the pathomorphological workup of the resection specimens.

**Electronic supplementary material:**

The online version of this article (doi:10.1186/s12957-015-0572-z) contains supplementary material, which is available to authorized users.

## Background

Although neoadjuvant treatment of rectal cancer is a routine procedure, some clinicopathological aspects of neoadjuvant treatment remain difficult, even so many years after its introduction. Namely, the problem of making robust judgments on tumor regression by surgical pathologists has not been solved satisfactorily. Traditionally, histopathologists grade regression of cancer after neoadjuvant therapy by subjective assessment of residual tumor and features of the adjacent stroma. Current systems are variations on one of two main themes: by one approach (for example, the Dworak system [1]) wherein pathologists assess residual vital tumor in relation to that part of the surrounding stroma or extracellular matrix with characteristic features of regressing tumor; by the other approach, pathologists make a mental sketch of a hypothetical ‘tumor bed’ and then estimate the percentage of vital remaining tumor in this region [2]. Whichever approach is chosen, regression grading is a subjective procedure and interobserver reproducibility is moderate to poor, at least in a multi-institutional setting [3], although better performance has been reported for a single-institution series [4]. Morphometric assessment of tumor regression has not been reported so far.

In most published studies, clinicopathological TNM stage is superior to or at least as important as the grade of tumor regression in predicting survival of rectal cancer patients. However, a dedicated histopathological workup of resection specimens could supply information of potential prognostic value beyond TNM. Specifically, histopathological features that may be worth paying attention to in this respect are: measuring depth of extramural infiltration, recording transgression of the lymph node capsule by metastatic tumor, as well as angioinvasion and perineural invasion. Status of the resection margin with measurement of distances is well appreciated as relevant [5].

In this study, we used our cases of clinically advanced, neoadjuvantly treated rectal cancers accessioned in the years 2000 to 2010 to test the prognostic potential of an assessment of tumor regression that relies on metric data and to compare these with conventional regression grading. Furthermore, in a study review, the histopathological features mentioned above were recorded and their impact on local recurrence was explored.

## Methods

### Composition of the study series and clinical data

This study includes all patients who underwent neoadjuvant long-term intensified chemoradiation for a clinically advanced, biopsy-proven adenocarcinoma of the rectum (0 to 16 cm from the anal verge) in the years 2000 to 2010 in the Department of Radiotherapy, Rostock University and who subsequently were operated in the Department of Surgery, Rostock University; histopathological examination of the resection specimens was done in the Institute of Pathology, Rostock University. Seventy-six patients were men, 28 were women, age 21 to 79 years (mean 61.98 years, median 63.87 years). For pretherapeutic clinical staging, a clinical history of the patients was taken and patients had a clinical examination as well as rigid rectoscopy to determine the distance of the lowest tumor border from the anal verge, colonoscopy, endosonographic ultrasound and/or imaging studies of the pelvis (MRI or CT), a CT scan and/or ultrasound examination of the abdomen, liver function tests, and chest X-ray, or CT of the thorax; additional imaging studies were done in the case of suspected metastatic disease. Details of the neoadjuvant therapy and the surgical procedures have been published previously [6]. Briefly, patients received pelvic radiation of 1.8 Gy single dose five days a week, adding up to 50.4 Gy (years 2000 to 2002; *N* = 20) or 55.8 Gy (after 2002; *N* = 84); this was complemented by chemotherapy with 5-FU or capecetabine and irinotecan or oxaliplatin. Surgery was scheduled six weeks after neoadjuvant treatment and included a total mesorectal excision for cancers of the low (0 to < 6 cm) and middle (6 to <12 cm) rectum and a partial mesorectal excision for cancers of the upper rectum (12 to 16 cm).

Follow-up data were obtained by a systematic approach. A standardized questionnaire was circulated to the referring physicians/primary care physicians at regular intervals requesting information on vital status, local recurrence, and metachronous metastatic disease. In addition, clinical information from the computerized hospital database was integrated and a check on the patients’ vital status was made with the Berlin Central Cancer Registry where death certificates from all deaths throughout Germany are recorded. Mean and median follow-up times were 79.4 and 78.5 months, respectively. The study was approved by the Medical Ethical Committee of Rostock University.

### Pathomorphological examination of the resection specimens

Surgical resection specimens were opened along the anterior aspect of the rectum and fixed overnight in buffered formalin. Dissections were carried out according to a standard in-house protocol that included blocking of the complete tumor/residual lesion if below 3 cm in the largest diameter and generous blocking in the remaining cases (8.62 and 8.00 paraffin-blocks as mean and median, respectively). Distances between the tumor and the circumferential margins were measured; paraffin-blocks representing extramural residual tumor and the inked circumferential margin were specifically taken if distances below 0.5 cm were observed on dissection. Completeness of the mesorectum was assessed (starting in 2002) and was scored as good or moderate (grading as in reference [7]; analogous to Mercury 1 or 2) in 90.4% of the cases evaluated for this feature and poor (Mercury 3) for the remaining 9.6%. Mesorectal fat was cleared in acetone overnight to allow maximum harvest of lymph nodes (mean 15.17, median 15, range 4 to 43). Surgical pathology reports typed and staged the tumors according to the TNM system. The first author made 65.4% of the dissections and initial pathology reports; the remaining reports were in equal proportions by three other consultant pathologists.

At the beginning of the study, the archived slides were retrieved and a study review was made, with request for additional sections from the archived paraffin-blocks if considered appropriate. This study review had three objectives: First, to confirm (or modify) the initial pathology reports and to add information on angioinvasion (L- and V-status) and perineural invasion (Pn-status) if missing. Second, to record the following additional histomorphological features:

1. Regression grading as published by Dworak. [1]
2. Maximum extramural extension of vital/non-regressed tumor, measured in millimeters and classified as absent, early (<3 mm), progressed (3 to 10 mm), or deep (>10 mm).
3. Minimum distance to the circumferential resection margin (measured in millimeters) and classified as >3 mm, 1 to 3 mm, or <1 mm.
4. Transgression of the lymph node capsule by metastatic tumor.

Third, in the course of the study review for each case, the H&E stained section that contained a full transection of the lesion and the maximum of vital tumor was selected for morphometric study.

### Morphometric studies

The slides selected during the study review were scanned with a Mirax slide scanner (Zeiss, Jena, Germany) and converted to digital images in JPEG format that allowed further processing with ImageJ (public domain software at http://rsb.info.nih.gov/nih-image) for morphometry. In these digital images, a ‘tumor bed’ was delineated as the area of interest (AOI). These AOIs contained the complete area of vital and/or regressed tumor from immediately below the superficial ulceration or re-epithelialized surface to the deepest border that in a given section could be traced by a continuous line. Avital/regressing tumor was defined by fibrotic tissue with histiocytic inflammatory infiltration that replaced the preexisting muscularis propria or extramural fat, often interspersed with areas of necrosis or dystrophic calcifications or mucin ‘lakes’; pure fibrosis which may well be radiation fibrosis only was not taken as regressing tumor.

**Figure 1:**
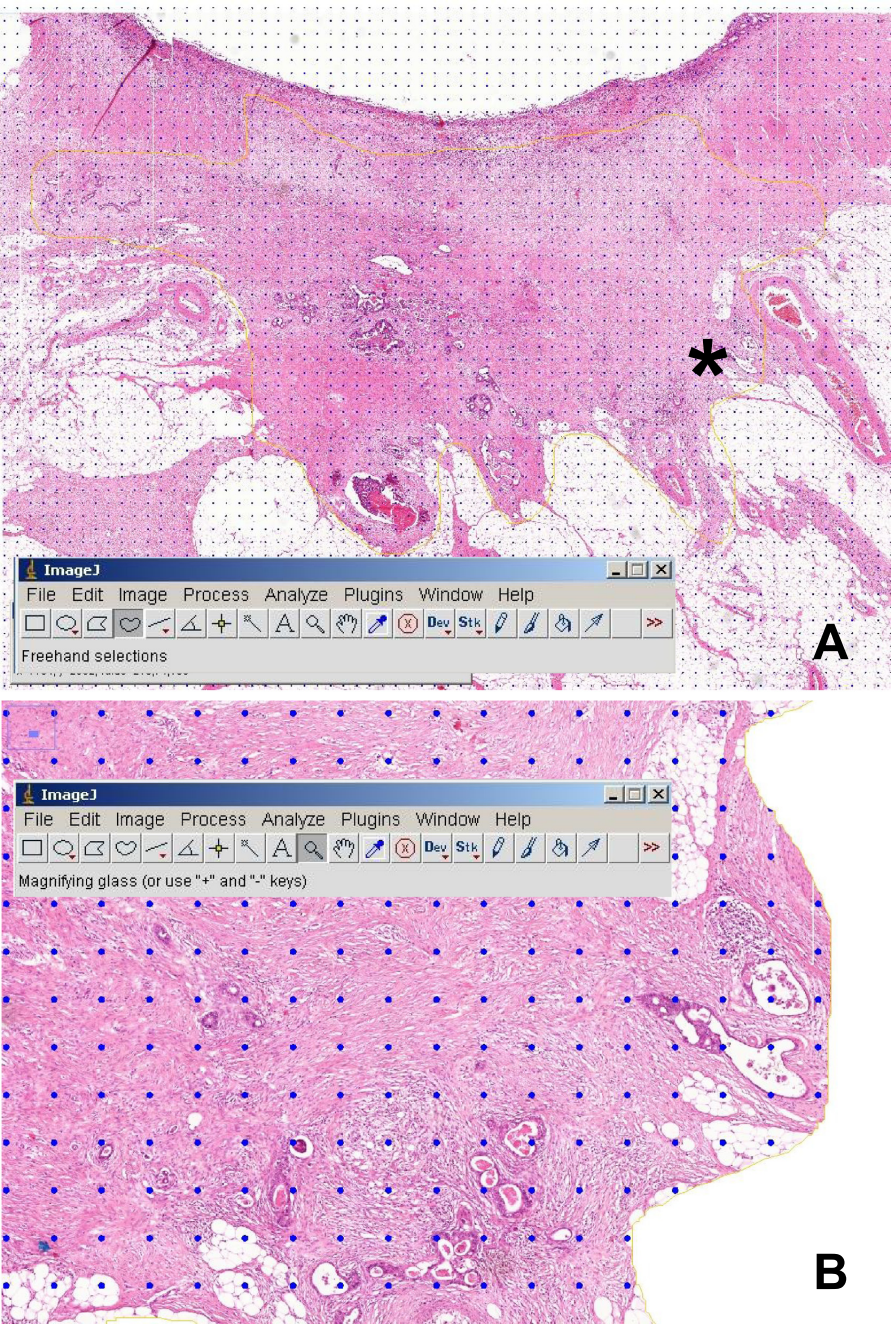
Screenshots illustrating the morphometric evaluation of scanned slides using ImageJ (see toolbars). **(A)** Panoramic view of the scanned slide. Note the area of interest delineated with a yellow line using the free-hand tool. Grid overlay for point counting has already been done. The asterisk indicates the area enlarged in **(B)** where grid points co-localizing with tumor cells (‘hits’) can be counted.

For this ‘tumor bed’, a numeric index (tumor area fraction (TAF)) was calculated based on a point counting method as follows (see Figure 1 for some exemplary screenshot images taken during the procedure):

1. Delineate an area of interest (AOI) representing vital and/or regressed tumor (the ‘tumor bed’) with the free-hand tool; clear the outside (ImageJ pull-down: edit ➔ clear outside).
2. Measure the number of pixels contained in this AOI (ImageJ pull-down: measure).
3. Place a ‘grid’ made up of points over the AOI (ImageJ pull-down: plugins ➔ particle analysis ➔ grid; specify grid characteristics as implemented with the selection menu); space points at 10.000 to 2,500 per pixel^2^, depending on the amount of vital tumor.
4. Calculate the total number of grid points contained in the AOI with the information from numbers 2 and 3.
5. Count the number of grid points co-localizing with vital tumor cells (‘hits’) by systematically moving through the image; magnify as appropriate with the ImageJ magnifying tool to allow discrimination of tumor from the stroma.
6. Divide the number of ‘hits’ by the total number of points contained in the AOI to obtain the TAF.

Resolution of histological detail afforded by this method is good, and the evaluations could be made with confidence. The evaluations were made by the pathologist among the authors (FP); except for a selected set of training cases (*N* = 10) to test precision/reproducibility of the method, for each case, the evaluation was performed once. To test precision/reproducibility of the method, a re-evaluation was done with ten of the cases. The re-evaluations included delineation of the ‘tumor bed’. A scatter graph plotting first vs. second evaluation and a Bland-Altman plot are provided as Additional file 1: Figure S1 and Additional file 2: Figure S2.

TAFs obtained for tumors of the study series were compared to cases from a control series of colorectal carcinomas that had not undergone preoperative treatment. This control series consisted of a total of 45 cases taken from the archives: 15 colorectal non-mucinous adenocarcinomas with an invasive border of the expansive type, 15 non-mucinous adenocarcinomas with an invasive border of the infiltrative type (see reference [8] for details on invasive margin typing), and 15 mucinous carcinomas.

### Statistical evaluations

All data were entered into a SPSS data bank (IBM SPSS Statistics 22). Relative risks for histopathological features for local recurrence were calculated from cross tabulations of classified data. Survival analyses were carried out according to the Kaplan-Meier method and significance testing was done by the log rank test.

## Results

### Clinicopathological features

This study includes 104 patients fulfilling the inclusion criteria specified above. Clinical data and the pathological data collected during the study review are summarized in Table 1. Complete regression of the primary tumor (ypT0) was seen in nine of the cases (Dworak regression grade (DRG) 4, 8.7%), although there remained vital nodal metastases in one case (that is, stage III disease) and distant metastases in two cases (stage IV disease). Regression of most of the primary tumor (DRG 3) was observed in another 17 cases (16.3%). According to a widely accepted suggestion [9], cases with a complete or near total regression can be combined in the category of ‘major regression’. This totalled at 24.9% in this series.

**Table 1:** **Clinical and pathological data**

	**DRG0**	**DRG1**	**DRG2**	**DRG3**	**DRG4**
Tumor location					
Upper rectum	0	2	3	0	0
Middle rectum	1	15	20	6	2
Lower rectum	0	13	24	11	7
Type of surgery					
Anterior resection	1	24	32	8	7
Amputation	0	6	15	9	2
UICC TNM stage					
Stage 0	0	0	0	0	6
Stage I	0	6	9	6	0
Stage II	0	8	8	5	0
Stage III	1	13	17	3	1
Stage IV	0	3	13	3	2
Depth of infiltration by ypT					
ypT0	0	0	0	0	9
ypT1	0	1	1	4	0
ypT2	0	6	12	6	0
ypT3	1	21	34	7	0
ypT4	0	2	0	0	0
Depth of extramural extension, vital tumor					
None	0	7	13	10	9
Early (<3 mm)	0	2	10	5	0
Progressed (3 to 10 mm)	1	16	19	2	0
Deep (>10 mm)	0	5	5	0	0
Nodal status (ypN)					
ypN0	0	15	20	13	7
ypN1	1	7	17	3	1
ypN2	0	8	10	1	1
Lymphatic spread (L)					
Absent	0	25	43	17	9
Present	1	5	4	0	0
Venous angioinvasion (V)					
Absent	0	21	37	15	8
Present	1	9	10	2	1
Perineural spread (Pn)					
Absent	0	24	41	17	8
Present	1	6	6	0	1
Distance towards CRM^a,b^					
>3 mm	1	22	35	17	8
1 to 3 mm	0	4	5	0	0
<1 mm	0	4	6	0	1
Local recurrence					
Negative	1	28	40	14	9
Positive	0	2	6	2	0
Death of disease					
Negative	0	23	22	12	8
Positive	1	7	24	4	1

^a^CRM, circumferential resection margin; ^b^Measurement not possible for technical reasons in one case. DRG, Dworak regression grade.

### Morphometric studies

**Figure 2:**
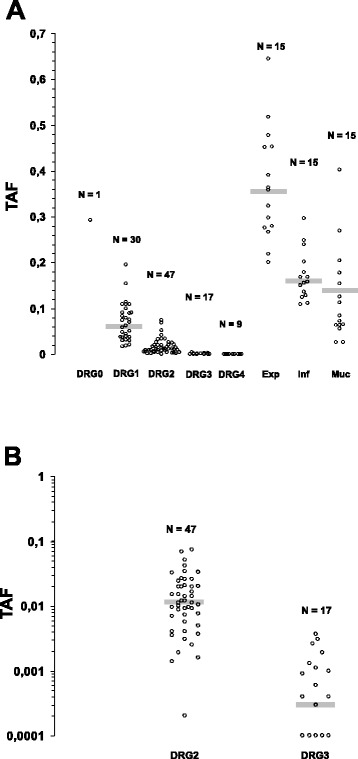
Scatter plot of TAFs. In **(A)** all TAFs obtained in this study are grouped by Dworak regression grades (study cases) or histotypes/type of invasive margin (control cases). Gray bars represent the medians within the groups. In **(B)** TAFs for cases DRG2 and DRG3 are plotted on a logarithmic scale. This expansion of the data overlay between groups is further appreciated. Note that assigning regression grade DRG2 to one tumor with a TAF well below the rest was due to a fairly large albeit single remaining vital tumor complex. Exp, expansive; inf, infiltrative; muc, mucinous; TAF, tumor area fractions; DRG, Dworak regression grade.

TAFs were determined for the rectal cancers and the control cases, they are plotted in Figure 2. In this figure, cases are grouped by DRG and the control cases as non-mucinous adenocarcinomas (with invasive margins of the expansive or infiltrative type) or mucinous adenocarcinomas. As can be gleaned from this figure, a clear-cut effect of neoadjuvant treatment in terms of reduced TAFs as compared to the control cases was observed for all rectal cancers classified as DRG1 to 4; although, notably, the TAFs determined for about half of the mucinous adenocarcinomas were in the range of values observed for rectal cancers classified as DRG1 or DRG2. Furthermore, it should also be noted that TAFs of non-mucinous adenocarcinomas with an invasive border of the infiltrative type were well below the TAFs of expansive type carcinomas.

TAFs were distributed continuously towards TAF = 0 (that is, complete regression) as is well appreciated in the plot in Figure 2a and by plotting the data on a log scale for the more regressed cases (DRG2 and DRG3, Figure 2b). Notably, TAFs overlapped considerably between DRG groups. Thus, regression grading in surgical pathology essentially amounts to an arbitrary division of a ‘morphological continuum’.

### Prognostic role of tumor regression and other histopathological features

Two of the 104 patients included in the histopathological and morphometric studies died of perioperative complications and were excluded from the survival analyses. Local recurrence was observed in 10 of the remaining 102 patients, two and four with additional distant metastases at first presentation or during follow-up, respectively. In two cases, local recurrence was proven by biopsy; in eight cases the diagnoses were made on imaging studies. As can be seen in Table 2, where details for these patients’ tumors are listed, all patients with local recurrence during follow-up had rectal cancer at a well advanced stage, frequently with a combination of several risk factors. Relative risks for local recurrence were calculated from cross tabulations and were found to be significant for the following risk factors: perineural infiltration (Pn1) and capsule transgressing mesorectal lymph node metastases and/or mesorectal tumor deposits. By contrast, tumor regression, extension of the tumor towards the circumferential resection margin (R1), lymphatic spread (L1), or venous angioinvasion (V1) as well as location of the tumor in the lower rectum or amputation as operative procedure were not found to be significant risk factors for local recurrence in this series. Furthermore, depth of extramural extension and TAF did not differ significantly between cases with and without local recurrence (*T*-test).

**Table 2:** **Clinicopathological features of cases with local recurrence**

**OP**	**Mesorectum** ^**a**^	**UICC stage**	**MET** ^**b**^	**TAF (DRG)**	**ypT**	**Extramural spread (mm)**	**ypN**	**Sat** ^**c**^	**L**	**V**	**Pn**	**R**
AR	ND	3	0	.0001 (3)	2	0	1	None	0	0	0	>3 mm
AR	Good	3	1	.0703 (1)	3	6	2	Positive	0	1	1	>3 mm
AR	ND	3	0	.0185 (1)	3	10	2	Positive	0	1	1	0 mm
Amp	Good	3	0	.0095 (2)	2	2	1	Positive	0	1	1	>3 mm
AR	Good	3	1	.0092 (2)	3	5	1	Positive	0	0	0	>3 mm
AR	Good	3	1	.0328 (2)	3	7	2^d^	Positive	1	0	1	<1 mm
AR	Good	3	1	.0040 (2)	3	5	2	Positive	0	0	0	>3 mm
Amp	Moderate	3	0	.0514 (2)	3	15	1	None	0	1	1	>3 mm
Amp	Good	4	NA	.0003 (3)	1	0	1	None	0	0	0	>3 mm
AR	Good	4	NA	.0140 (2)	3	10	1	None	0	0	0	>3 mm

^a^State of the mesorectum, analogous to the Mercury classification; ^b^Distant metastases during follow-up; ^c^Sat, vital satellite nodules; ^d^with extranodal spread of vital tumor. NA, not applicable; ND, no data.

**Figure 3:**
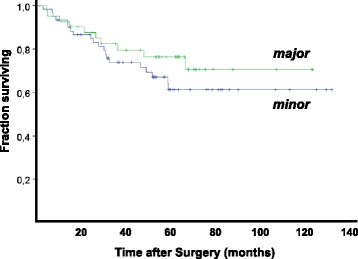
Kaplan-Meier survival curves with death of disease as clinical end-point and stratification according to TAFs. Tumors with TAFs below the 20th percentile were classified as major responders; the remainder as minor responders.

During follow-up, death of disease was observed for 37 of the 102 patients. Kaplan-Meier survival curves with extent of regression as prognostic factor are plotted in Figure 3. In this analysis, the 20% of the cases with lowest TAFs (≤20th percentile) were defined as having a major regression, the remainder as minor responders. As can be seen in Figure 3, survival for patients with major regressions were better, although significance by log rank test was not reached (*P* = 0,056). Defining DRGs 4 and 5 as major responders a similar result was observed, again not significant by log rank test. TNM stage was a strong and statistically significant prognosticator.

## Discussion

High interobserver variability is a drawback of current systems for grading tumor regression of neoadjuvantly treated rectal cancer [3], prompting this study to try for a more objective approach. We employed a traditional point counting method coupled with modern electronic image processing. Scanned sections were made available for virtual microscopy, an area corresponding to the ‘tumor bed’ was selected, measured, and covered with a grid for counting ‘hits’ on vital tumor that allowed calculating a TAF. Essentially, the procedure starts from the idea that also underlies regression grading approaches that make estimates of vital remaining tumor [2], yet by making measurements instead of subjective assessment of residual viable tumor, it goes beyond in two important aspects. First, precision and reduced subjectivity in these evaluations are an advantage. The second advantage of this morphometric approach is that it supplies numeric data which are suitable for additional exploratory analyses. However, doubtless this method is not completely without an arbitrary/subjective element. Namely, there remains the selection of the slide to study by morphometry, a well known confounder in regression grading [4]. The guideline applied here was to select the slide with the maximum burden of vital tumor. Furthermore, delineation of the ‘tumor bed’ remains a subjective procedure although reproducibility appeared not to be compromised much when repeat evaluations were made (see Additional file 1: Figure S1 and Additional file 2: Figure S2).

Plotting the numeric tumor regression data of the cases included in this study gives an easy answer to why pathologists have failed to conceive a regression grading system endowed with high interobserver reproducibility; this can be appreciated almost intuitively in the resulting Figure 2: the amount of residual tumor after neoadjuvant chemoradiation determined for a given case can fall anywhere within a broad continuous range. With the exception of cases with complete regression (8.7% in this series), indeed, there are not any natural cut points that allow meaningful distinctions between ‘grades’. Accordingly, overlap of TAFs between DRGs is considerable. From a tumor biological point of view this is not surprising, but as far as we are aware, this because of the lack of morphometric data has not been and could not be spelt out clearly. Another interesting aspect derives from these quantitative data when comparing them with the TAFs obtained for the control cases, particularly so if histotypes (adenocarcinoma vs. mucinous carcinoma) and phenotypes of invasion (expansive vs. infiltrative) are also taken into account. Only TAFs obtained for adenocarcinomas with an invasive border of the expansive type were well above the range of neoadjuvantly treated rectal cancers, but carcinomas with an invasive borders of the infiltrative type overlapped into the DRG1 group and mucinous carcinomas even into DRG2. Obviously, when examining the resection specimens, pathologists cannot know what type of cancer there was at the beginning of neoadjuvant chemoradiation. Thus, when trying to grade regression, apparently we are dealing with a situation where end-points differ considerably between cases, but the starting-points, unknown for each case, also are very different! By adding this underrecognized aspect into the account, regression grading is coming to light as really quite a conundrum.

What then could be a ‘major regression’? In a meaningful definition, assessment would have to rely on an objective and reproducible method, and a major regression thus determined would have to imply a favorable prognosis with significance. Two approaches to this issue can be found in published clinicopathological studies. By one approach, cases with complete regression are combined with those with minimal residual vital tumor (TRGs 1 and 2 in the Mandard system; DRGs 4 and 5 in the Dworak system). Clearly, this approach is compromised by problems with reproducibility/objectivity, as demonstrated by the interobserver studies [3] and explained by the morphometric data presented here. Nevertheless, in most [9-13] but not all [14,15] studies, a major regression thus defined was found to predict a favorable clinical course with statistical significance. Defining cases with TAFs ≤20th percentile as major responders or combining DRGs 4 and 5, we observed the same result, although statistical significance was not quite reached (as in references [14,15]). Notably, our attempt to use the numeric regression data for prognostication with a cutoff did not perform better than traditional regression grading. Lack of statistical significance in the present series may well have been due to the relatively low number of cases, similar to the studies by Pucciarelli *et al*. and Hav *et al*. [14,15]. Furthermore, our series included a fairly large proportion of patients with synchronous distant metastases that may be limiting in the clinical course and would not be targeted by the neoadjuvant therapy as efficiently as the primary tumor and its deposits in the pelvic region. By the other approach, major regressions are restricted to cases with complete sterilization of the tumor (see reference [16] for meta-analyses) At first sight, this may seem a very robust definition and a natural cutoff, vindicated further by the prognostic significance demonstrated by the meta-analyses. However, as pointed out by Bateman *et al*. [17], a very thorough and dedicated histopathological workup of the specimens is mandatory for this, giving rise to the mischievous thought that, in view of very high rates of complete responses in some of the series basing these meta-analyses, perhaps sufficient diagnostic rigor in the determinations of complete pathological responses had not been executed in all the studies. Taken together, *Pathological complete response: still a relevant end-point in rectal cancer?* is a legitimate question to ask [18].

It must be realized that in all the clinicopathological studies on tumor regression, TNM stage was found to be more important as a prognostic factor than tumor regression, and this was our observation, too. Thus, careful histopathologic workup of the specimens appears to be the most important issue. It may indeed even be worthwhile to invest more effort into this: in this series, for example, perineural invasion and capsule transgression of lymph node metastases/perirectal tumor deposits were found to be a strong adverse prognosticator for local recurrence. Similarly, tumor deposits (interpreted as tumor fragmentation as opposed to tumor shrinkage) were a significant negative histopathological factor in a recent study [15].

## Conclusions

In conclusion, we showed in this study that morphometric assessment of tumor regression is feasible, explaining why conventional regression grading is so difficult to perform. Furthermore, assessment of tumor regression, by subjective grading or morphometry, apparently does not convey major prognostic information, at least not convincingly beyond careful histopathological tumor staging. This observation casts doubt on expending too much effort on developing this aspect of the pathomorphological workup of the resection specimens.

## Additional files

Additional file 1: Figure S1. Scatter plot of TAFs obtained by re-evaluation of a selected set of cases. Additional file 2: Figure S2. Bland-Altman plot of re-evaluations. The dashed horizontal line in the middle signifies the mean of the differences; the dashed horizontal lines above and below signify +/− 2 s, respectively.
