# Supplementary figures and images for: Tumor regression in rectal cancer after intensified neoadjuvant chemoradiation: a morphometric and clinicopathological study
Source: World J Surg Oncol. 2015 Apr 21;13:155. doi: 10.1186/s12957-015-0572-z (PMC4415293; doi:10.1186/s12957-015-0572-z)

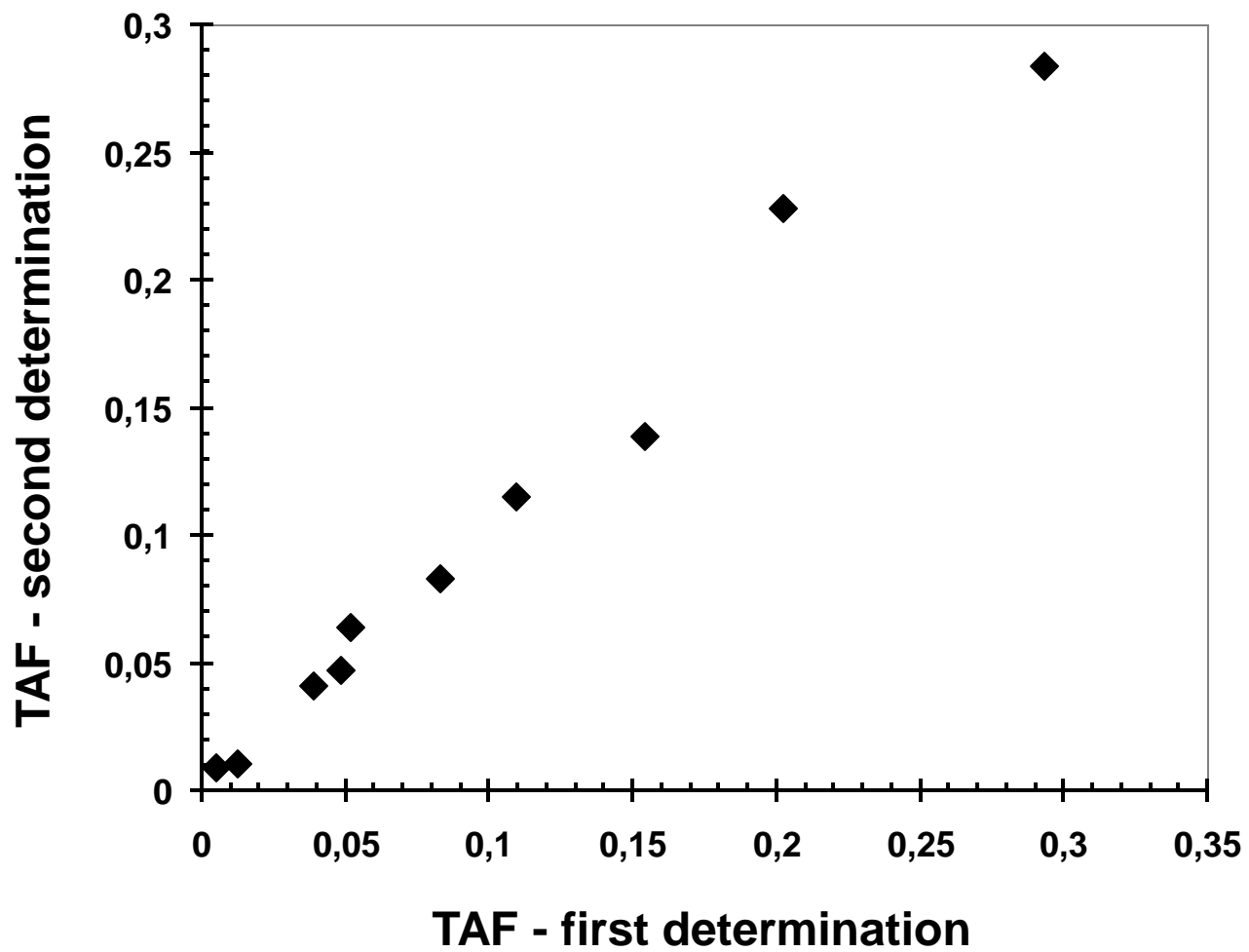

Supplement: Additional file 1: Figure S1. — Scatter plot of TAFs obtained by re-evaluation of a selected set of cases. [file 12957_2015_572_MOESM1_ESM.pdf]

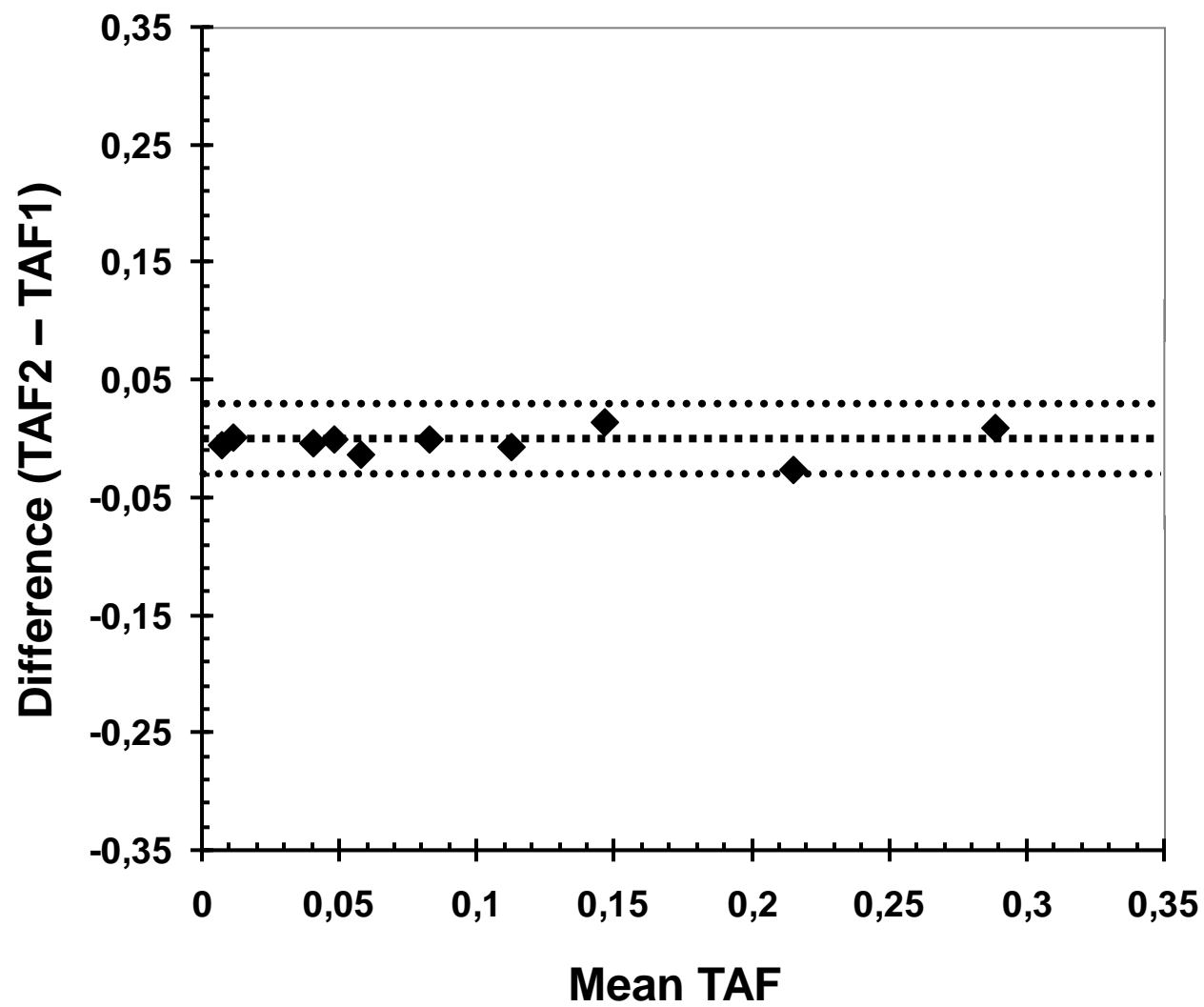

Supplement: Additional file 2: Figure S2. — Bland-Altman plot of re-evaluations. The dashed horizontal line in the middle signifies the mean of the differences; the dashed horizontal lines above and below signify +/− 2 s, respectively. [file 12957_2015_572_MOESM2_ESM.pdf]
